# Supplementary material for: Aberrant gene expression prediction across human tissues
Source: Nat Commun. 2025 Mar 29;16:3061. doi: 10.1038/s41467-025-58210-w (PMC11954926; doi:10.1038/s41467-025-58210-w)
Supplement: Supplementary file 1 — Supplementary Information [file 41467_2025_58210_MOESM1_ESM.pdf]

Supplementary information

Supplementary figures

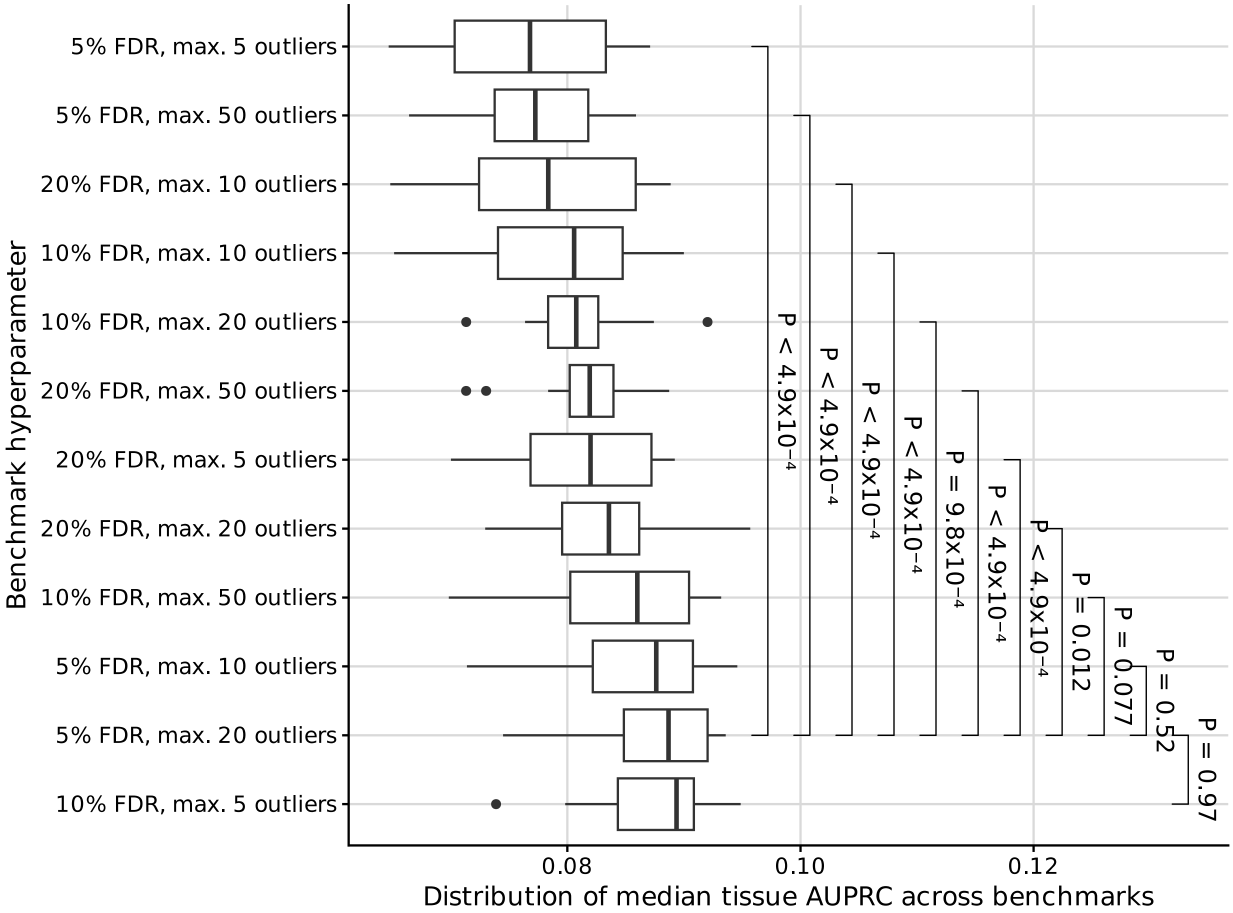

Supplementary Figure 1: OUTRIDER cutoff selection for the definition of the benchmark dataset. We evaluated various FDR thresholds (0.05, 0.1, and 0.2) and cutoffs on the number of outliers per sample (5, 10, 20, 50), both for training and evaluation. To this end, we trained simple models using previously established features (LOFTEE, CADD, VEP, and structural variants) to predict underexpression outliers with a LightGBM regression on the gene expression z-score (methods), ensuring that the cross-validation folds contain the same individuals in all settings. Performance was evaluated as the median area under the precision-recall curve across tissues under all these settings (boxplots, n=12 benchmark settings). Box label, sample size; Center line, median; box limits, first and third quartiles; whiskers span all data within 1.5 interquartile ranges of the lower and upper quartiles. The settings of the bottom two boxplots led to quasi-uniformly better models across all 12 benchmark settings used for evaluation. Among these two settings, we opted for 5% FDR and max. 20 outliers as it provided twice as many outliers for training.

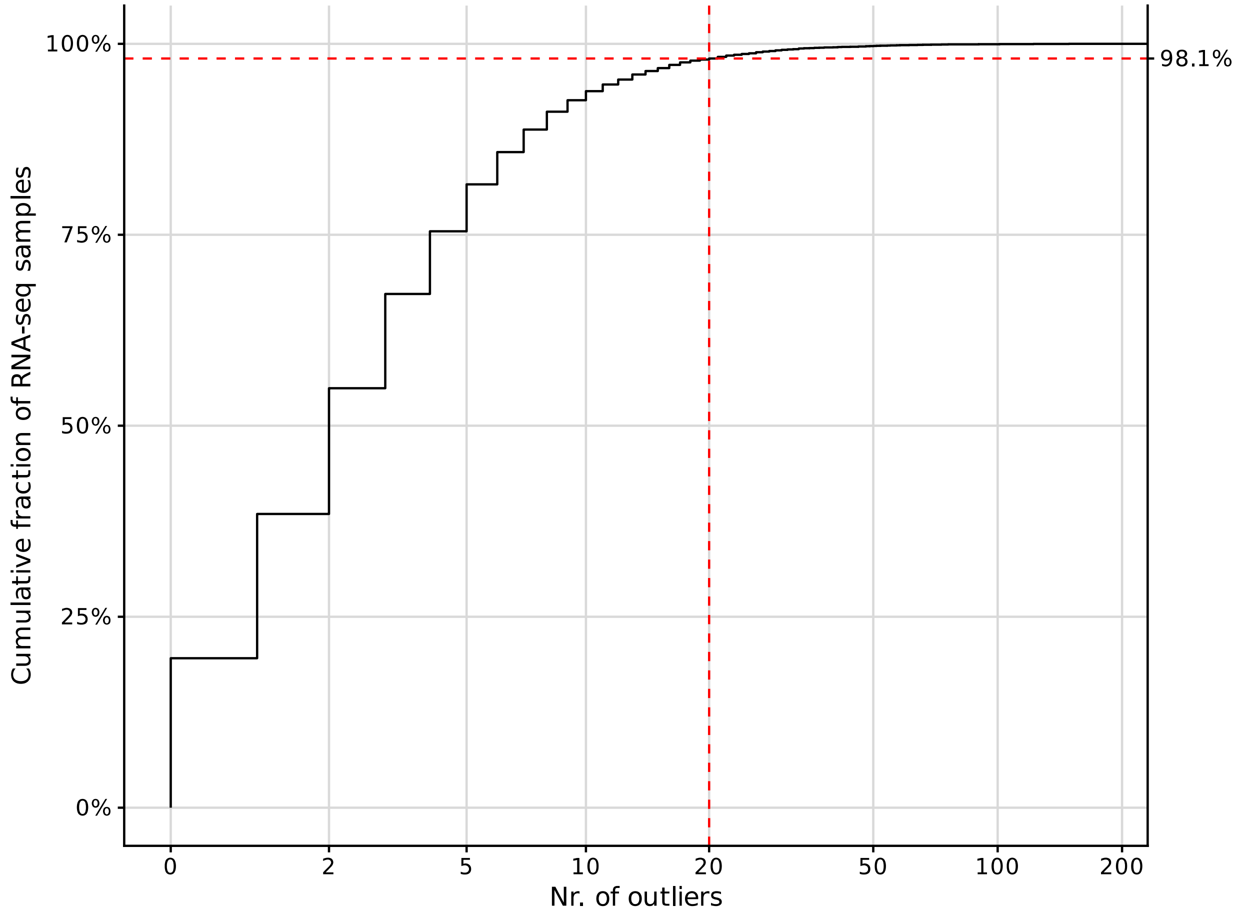

Supplementary Figure 2: Only 1.9% of GTEx transcriptome sequencing samples have more than 20 outliers. Cumulative fraction of 11,215 RNA-seq samples (y-axis) that have at most a given number of outliers (x-axis) in the GTEx dataset. The vertical dashed line denotes the 20 outlier cutoff, which 98.1% of samples passed (horizontal dashed line).

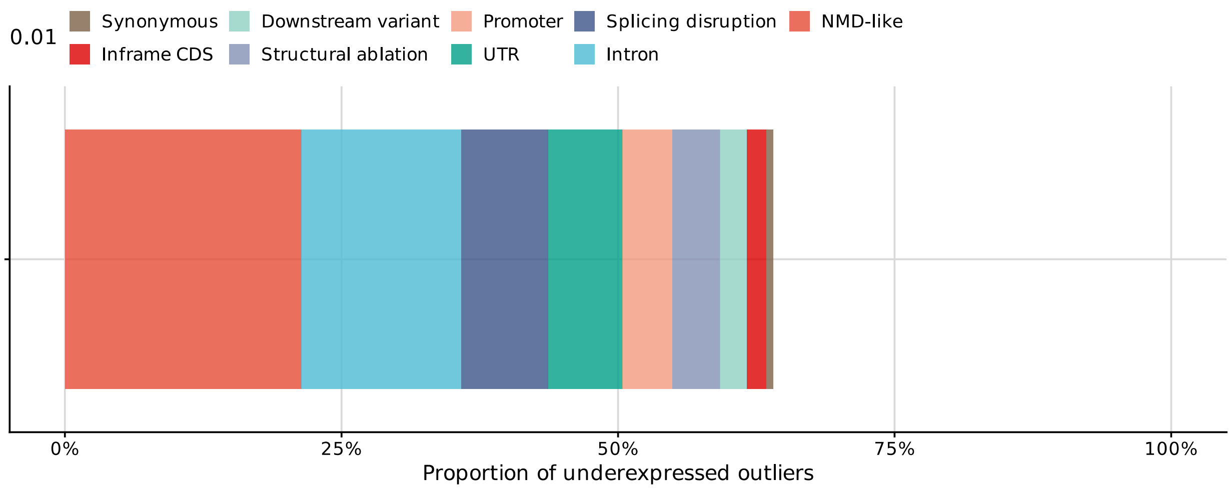

Supplementary Figure 3: Proportion of 11,200 underexpression outliers explained by different classes of variants. Each outlier is assigned to the most impactful consequence and therefore occurs only in one category (Methods).

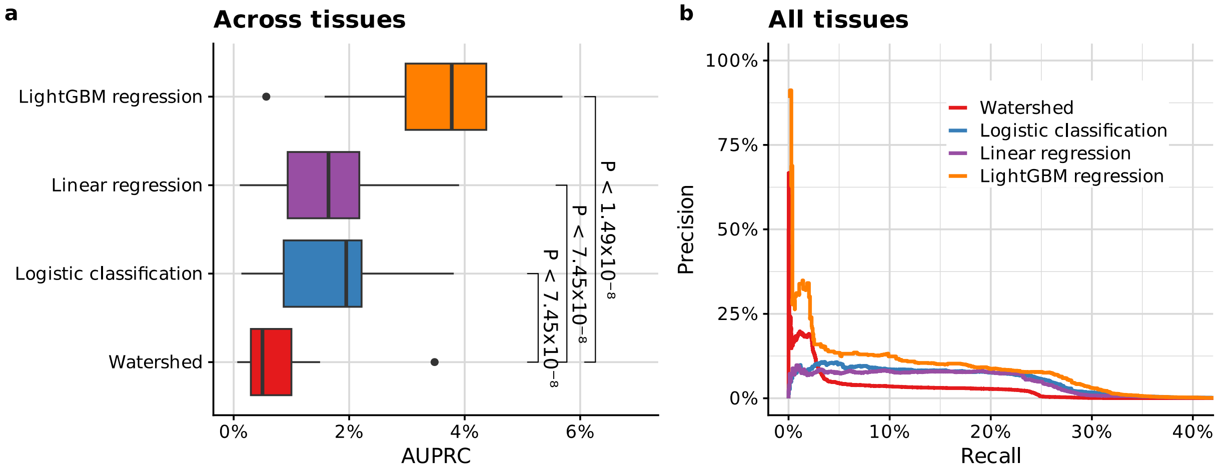

Supplementary Figure 4: Watershed model architecture is outperformed by classic machine learning methods. (a) Distribution of average precision (AUPRC) across 27 GTEx tissue types. P-values were obtained using the paired two-sided Wilcoxon test. Box label, sample size; Center line, median; box limits, first and third quartiles; whiskers span all data within 1.5 interquartile ranges of the lower and upper quartiles. (**b)** Precision-recall curve for all tissues combined.

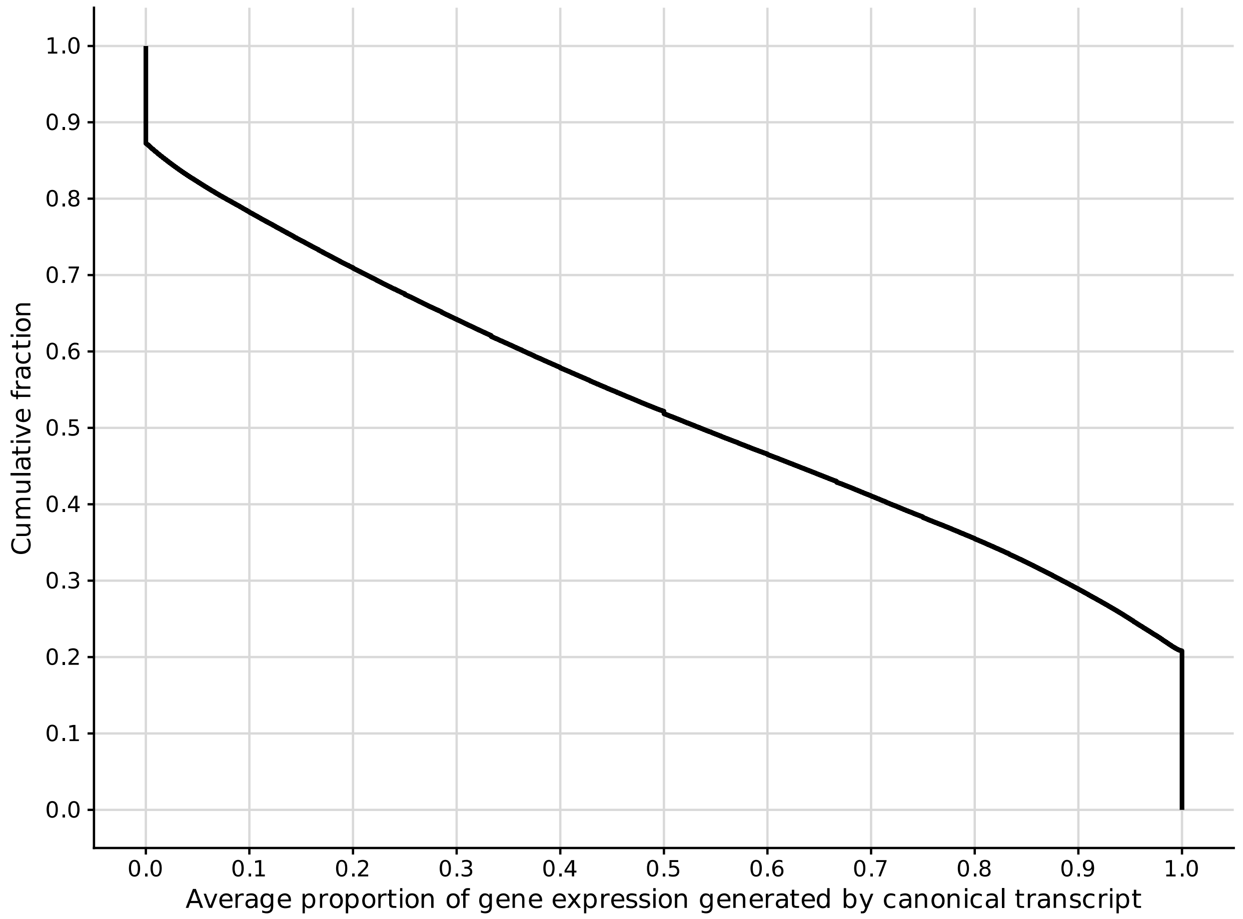

Supplementary Figure 5: **Cumulative fraction of genes (y-axis) for which the canonical transcript contributes to more than a given proportion (x-axis) to the total gene expression.** The data is aggregated over all tissues and restricted to the expressed genes per tissue, resulting in 984,312 transcript-tissue combinations. Canonical according to VEP.

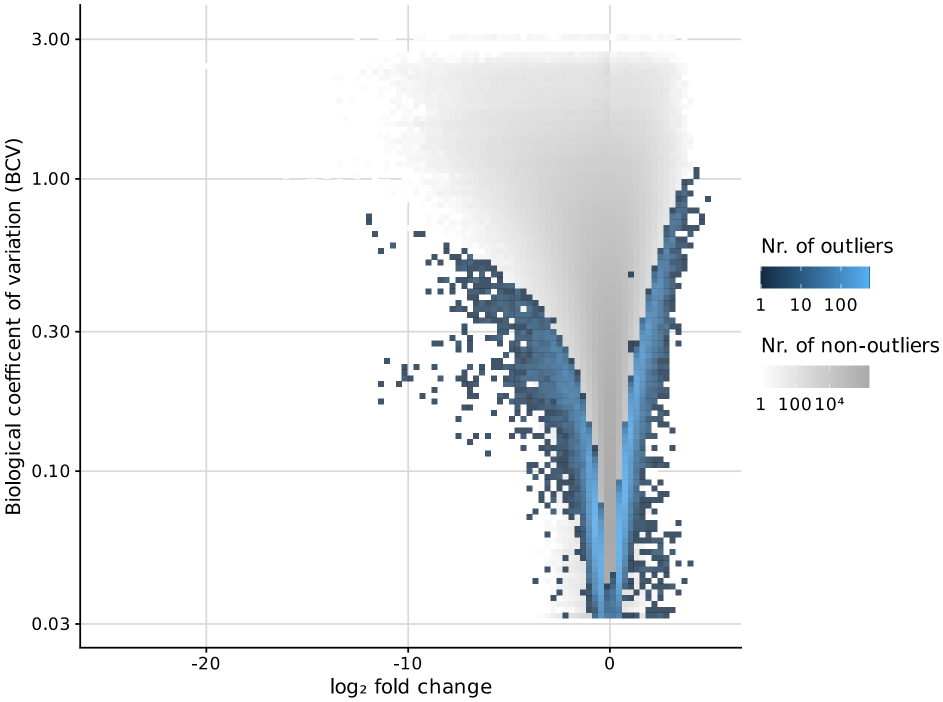

Supplementary Figure 6: **Biological coefficient of variation (BCV) against expression fold change across all genes and tissues.** The BCV is an estimate of the coefficient of variation of expression of a gene measured by RNA-seq read counts controlling for sampling noise. The larger the BCV of a gene in a tissue, the more variable it is across the GTEx population. BCV is used instead of standard deviation to account for sampling noise, which particularly affects low RNA-seq read counts (Methods). Highly variable genes require a larger fold change to be called an outlier.

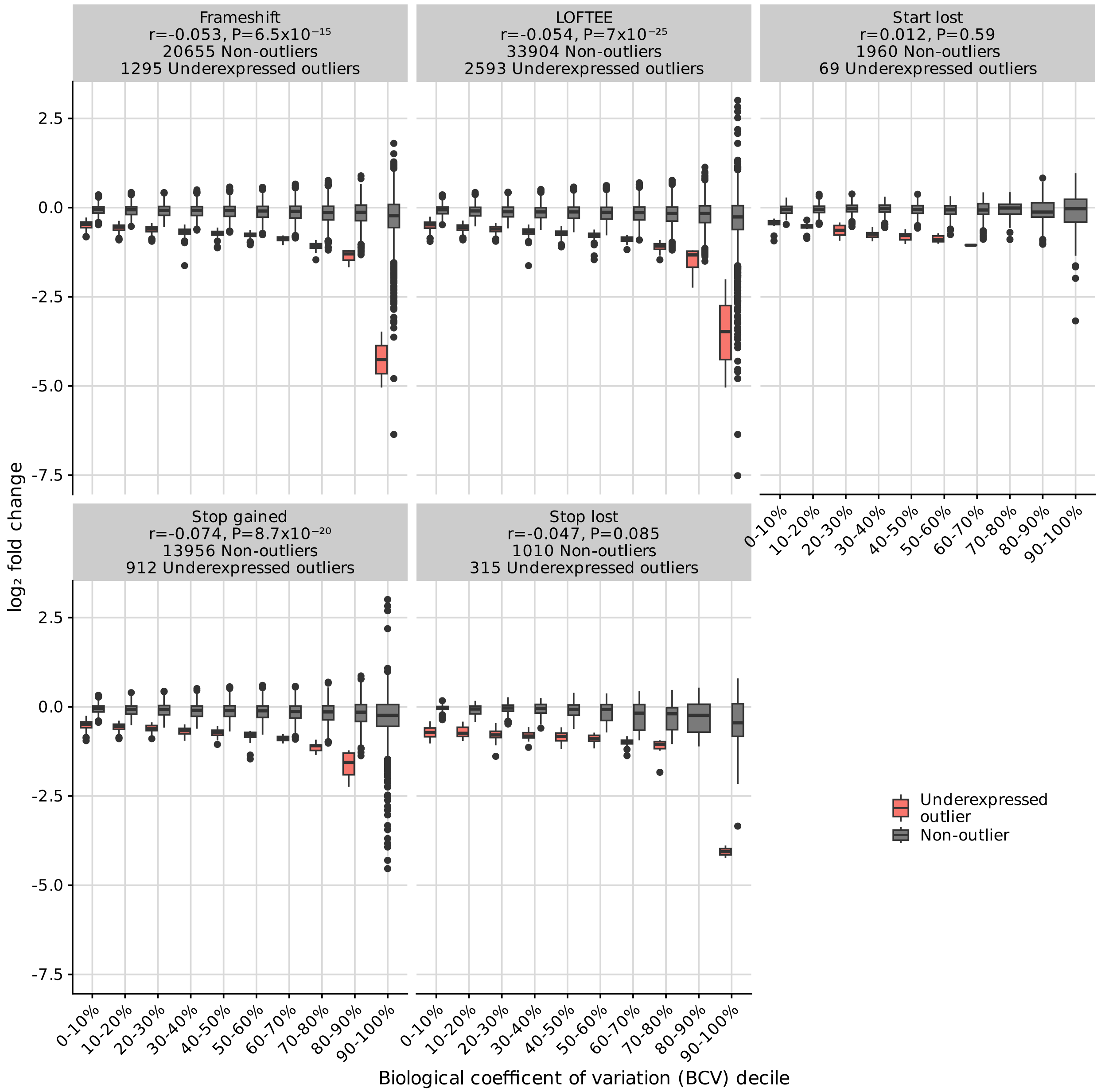

Supplementary Figure 7: **Distribution of gene expression fold changes among genes in different deciles of expression variability, given that the gene is affected by some rare variant consequence (e.g. frameshift, LOFTEE).** Center line, median; box limits, first and third quartiles; whiskers span all data within 1.5 interquartile ranges of the lower and upper quartiles. The higher the coefficient of variation, the larger the gene expression impact of the variants tends to be. If a gene has rare variants with multiple consequences, they will appear in all the corresponding panels.

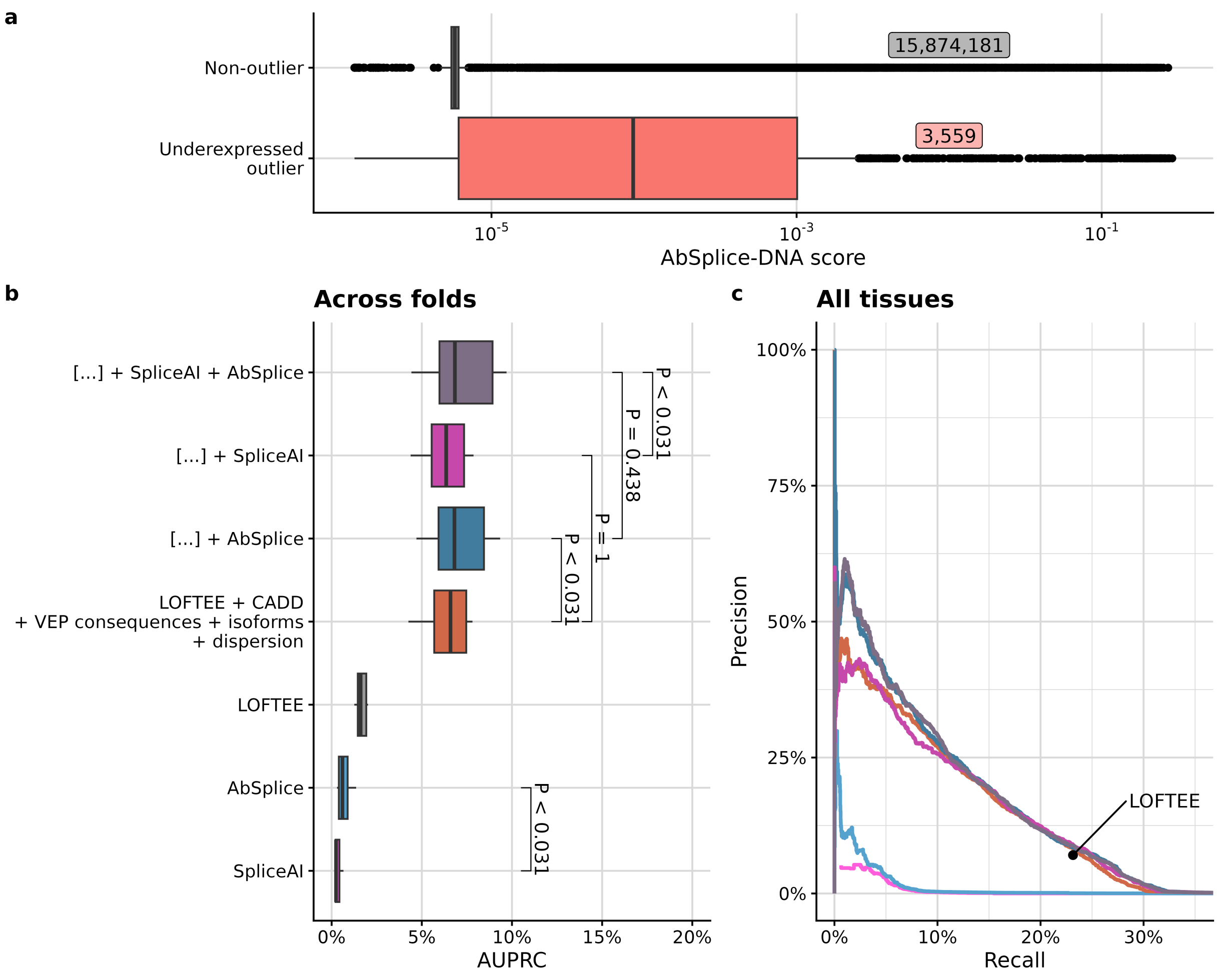

Supplementary Figure 8: **AbSplice-DNA improves the outlier prediction. (a)** Distribution of AbSplice-DNA scores in different outlier classes. The numbers of gene-sample pairs with AbSplice-DNA scores are labeled for each box. (**b)** Distribution of average precision (AUPRC) across 27 GTEx tissue types. *P*-values were obtained using the paired two-sided Wilcoxon test. Adding AbSplice-DNA as a feature significantly improves outlier prediction, whereas adding SpliceAI does not show any significant improvement. (**c)** Precision-recall curve for all tissues combined. Center line, median; box limits, first and third quartiles; whiskers span all data within 1.5 interquartile ranges of the lower and upper quartiles.

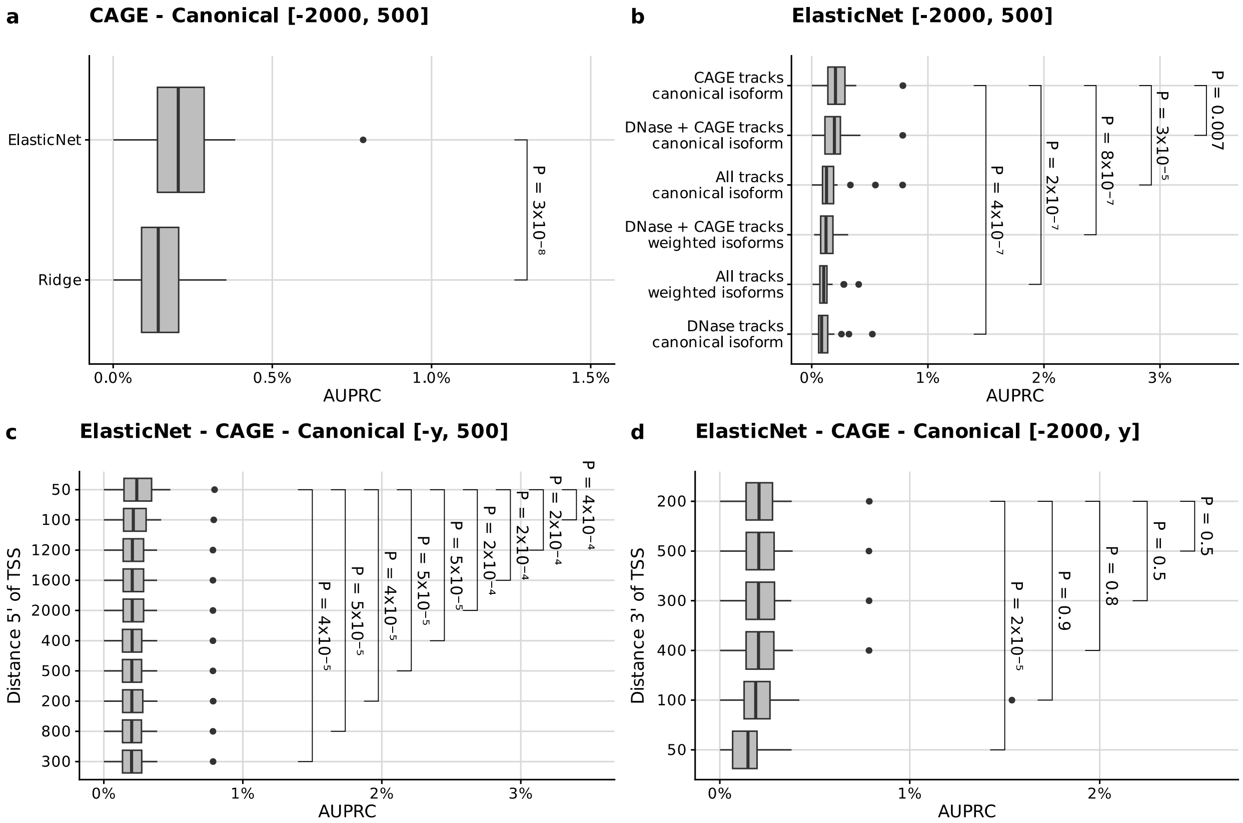

Supplementary Figure 9: Integration of Enformer. We trained models predicting gene expression averaged across individuals of GTEx for each of the 49 tissues from the reference genome. The models are all regularized linear regressions from some of the Enformer-predicted tracks (CAGE, DNAse, or all tracks) applied to either the canonical isoform only or to all isoforms weighted by their tissue-specific isoform composition. **(a)** Distribution of average precision (AUPRC) on underexpression prediction across 27 GTEx tissue types for elastic net (top) or ridge (bottom) canonical-isoform models using CAGE tracks. Variant effects were considered for variants within [-2000, +500] bp of the canonical transcription start site (TSS) and set to 0 otherwise. Elastic net, which is a generalization of ridge, outperformed ridge and was further used. **(b)** As in **a** for canonical-isoform and weighted-isoform elastic net models and including various Enformer-predicted tracks. Using CAGE predictions on the canonical TSS performs significantly better than other combinations and was further used. **(c)** as in **a** setting to 0 the variant effect predictions for variants beyond a certain distance (y-axis) of the TSS. Keeping effect predictions only for variants within 50 bp 5' of the TSS and setting effects to 0 otherwise resulted in the best median AUPRC. **(d)** as in **c** but 3' of the TSS. The 200 bp threshold led to the best median AUPRC. All box plots show the distribution of average precision (AUPRC) across 27 GTEx tissue types, sorted by the respective median AUPRCs. **For all boxplots:** Center line, median; box limits, first and third quartiles; whiskers span all data within 1.5 interquartile ranges of the lower and upper quartiles.*P*-values were obtained using the paired two-sided Wilcoxon test.

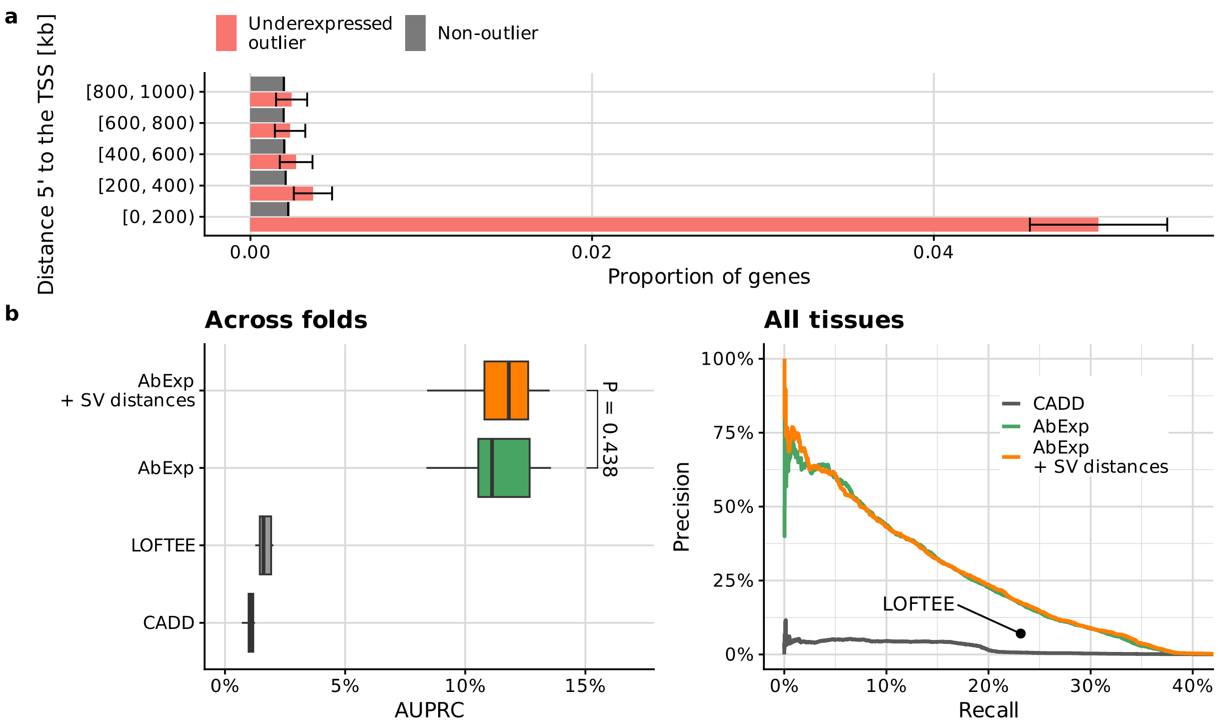

Supplementary Figure 10: Distal structural variants do not significantly improve underexpression outlier prediction. (a) Proportion of 11,200 underexpressed outliers (red), and 99,434,253 non-outliers (gray) with a structural variant within a certain distance 5’ of the transcription start site of the gene (rows, Methods). Error bars mark 95% binomial confidence intervals. **(b)** Left: Distribution of average precision (AUPRC) across 27 GTEx tissue types, using from bottom to top: CADD, LOFTEE, AbExp (which only has SVs within 5kb of the gene), and “AbExp + SV distances”, a model integrating the AbExp features and SVs up to 1 Mb 5’ of the gene stratified by distance bins as in **a)**. *P*-values were obtained using the paired two-sided Wilcoxon test. Center line, median; box limits, first and third quartiles; whiskers span all data within 1.5 interquartile ranges of the lower and upper quartiles. Right: Precision-recall curve for all tissues combined for the same models.

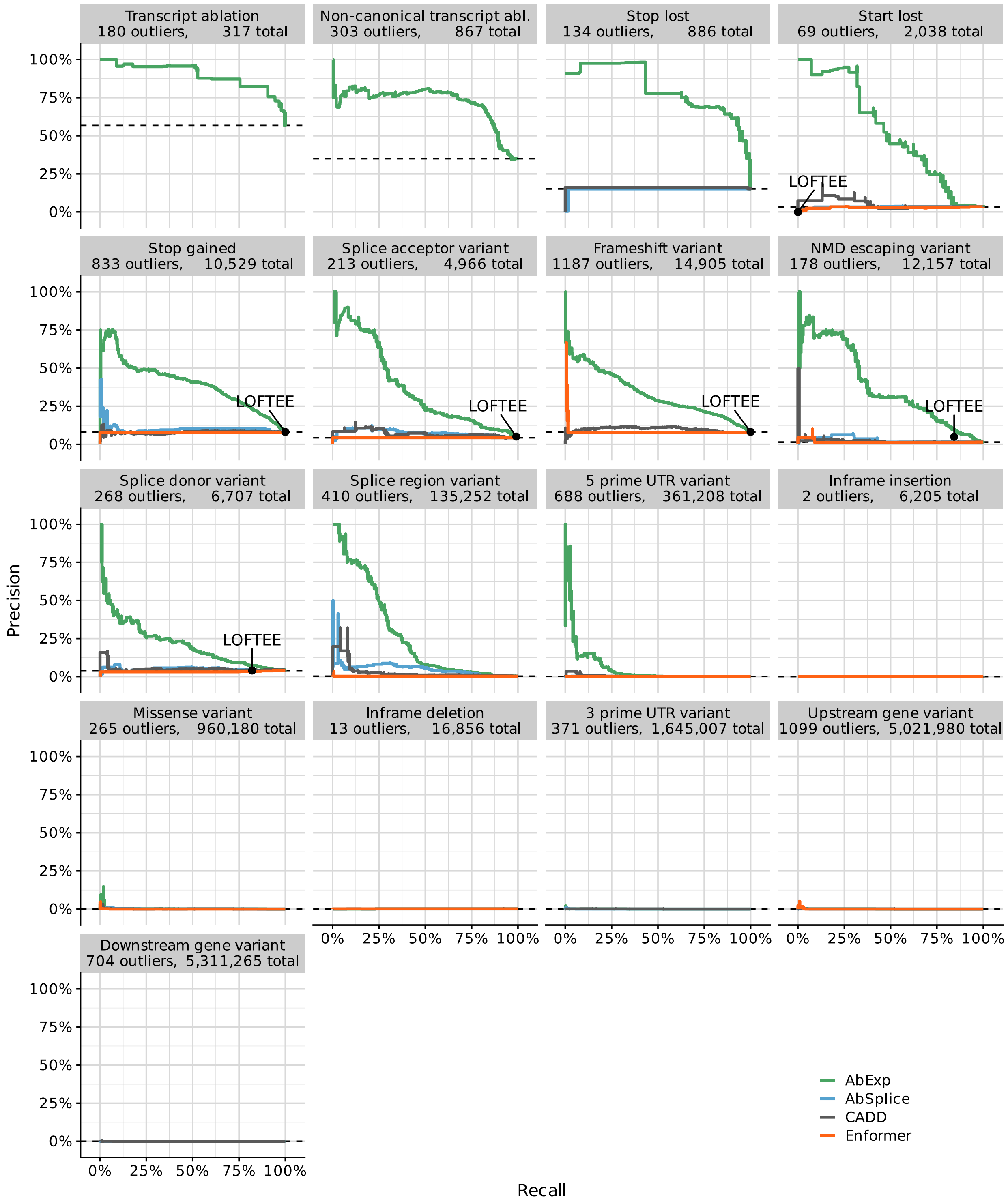

Supplementary Figure 11: **Precision-recall curves stratified by variant type for different models on the GTEx benchmark dataset.** Variant effect predictions were max-aggregated per gene, individual and tissue for all models. LOFTEE as a binary predictor is displayed as a single point. Horizontal dashed lines show the baseline precision for a predictor that classifies all genes as outliers.

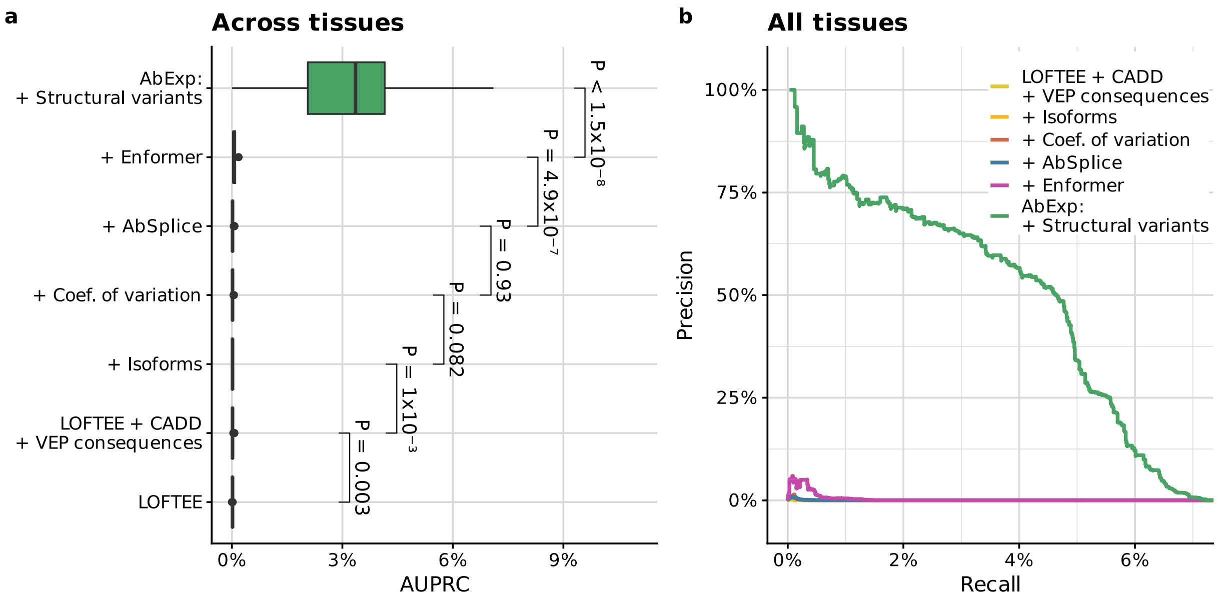

Supplementary Figure 12: Integrating Enformer and duplications significantly improves the overexpression outlier prediction. (a) Distribution of average precision (AUPRC) across 27 GTEx tissue types. *P*-values were obtained using the paired two-sided Wilcoxon test. Center line, median; box limits, first and third quartiles; whiskers span all data within 1.5 interquartile ranges of the lower and upper quartiles. **(b)** Precision-recall curve for all tissues combined.

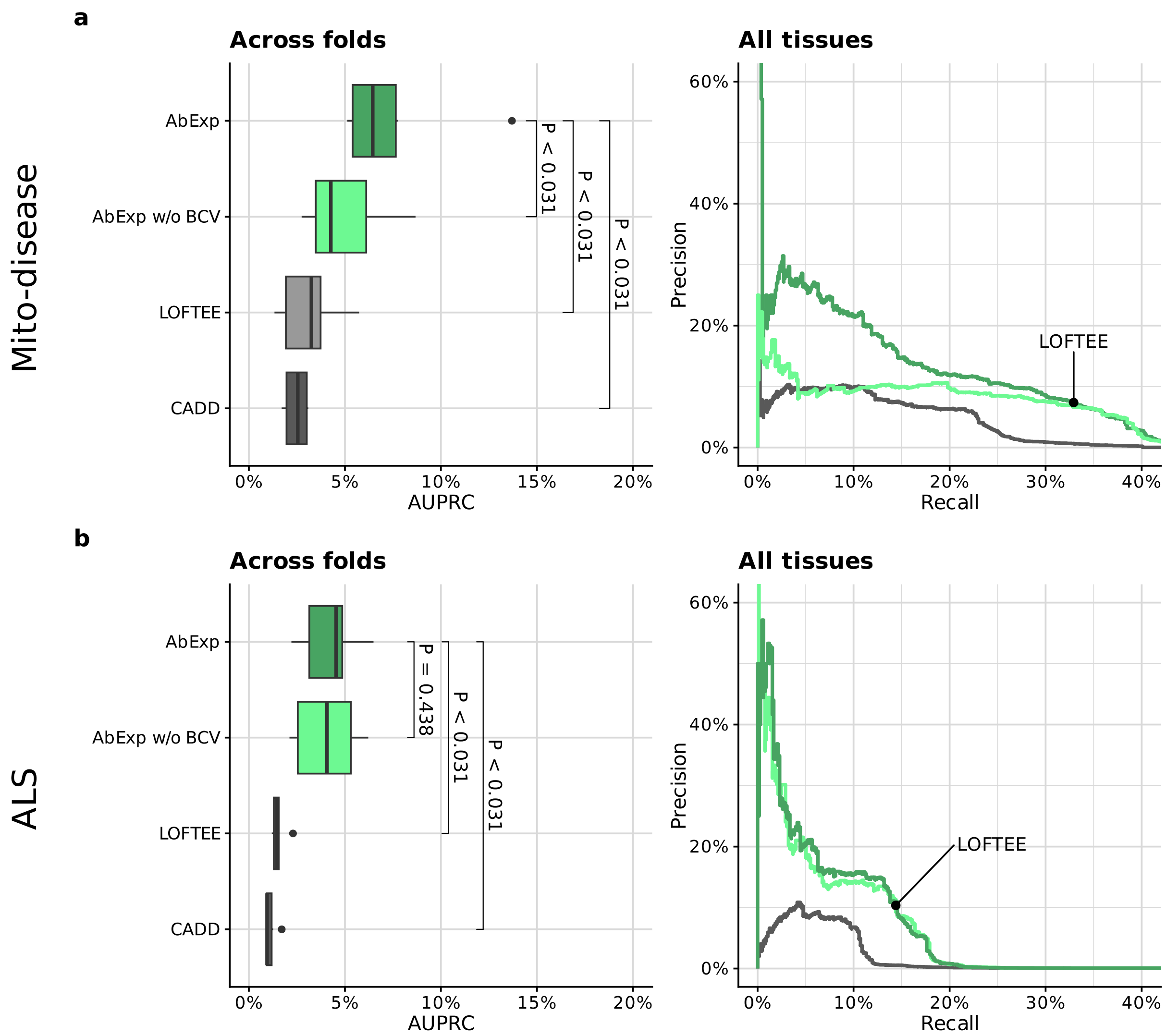

Supplementary Figure 13: Performance of AbExp on independent datasets. (a) Left: Distribution of average precision (AUPRC) across five cross-validation folds in the mito-disease dataset. *P*-values were obtained using a paired two-sided Wilcoxon test. Right: Precision-recall curve on the whole mito-disease dataset. LOFTEE as a binary predictor is shown as a single point. **(b)** Left: Distribution of average precision (AUPRC) across five cross-validation folds in the ALS dataset. *P*-values were obtained using a paired two-sided Wilcoxon test. Right: Precision-recall curve on the whole ALS dataset. LOFTEE as a binary predictor is shown as a single point. **All box plots:** Center line, median; box limits, first and third quartiles; whiskers span all data within 1.5 interquartile ranges of the lower and upper quartiles.

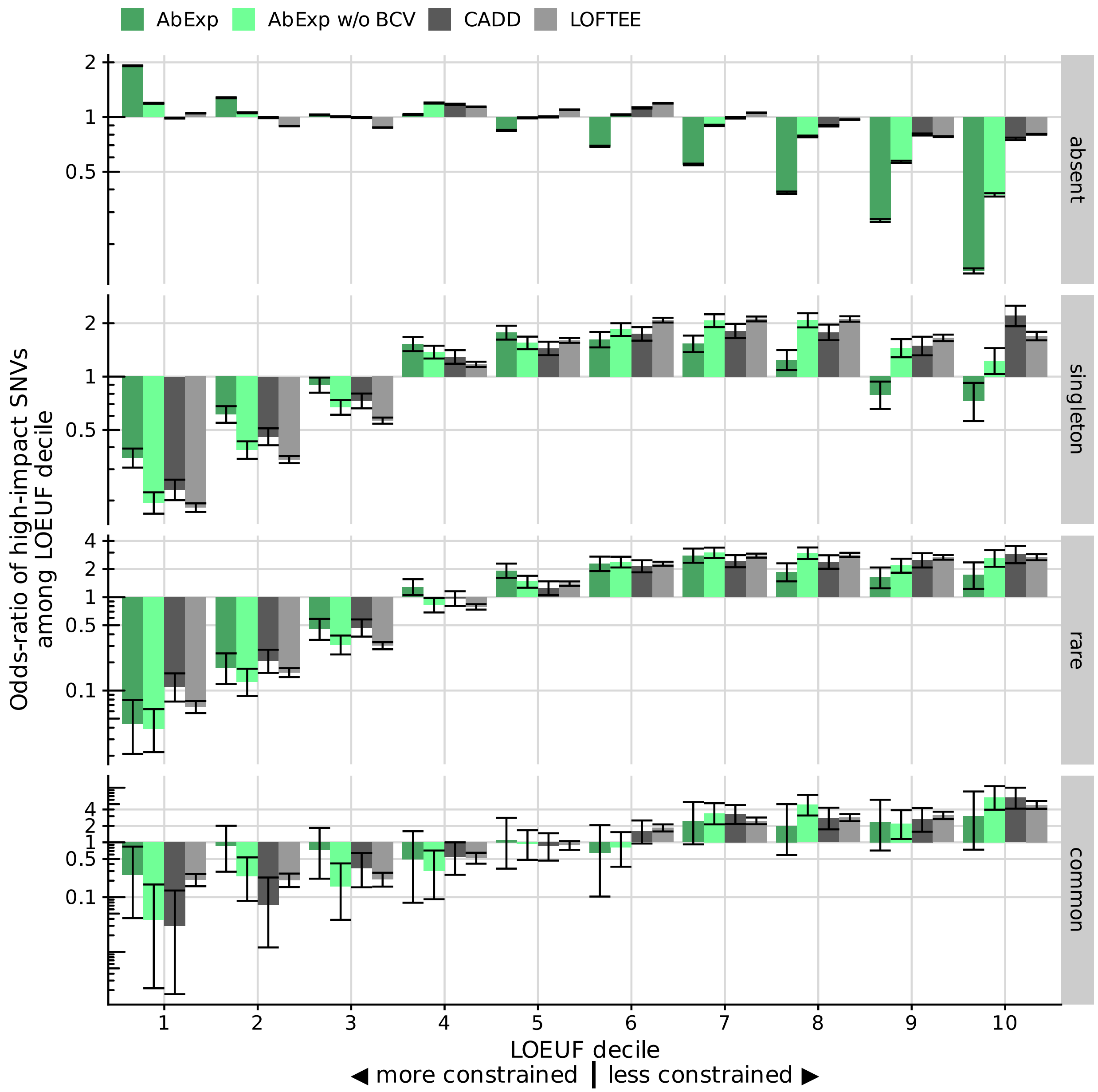

Supplementary Figure 14: Odds-ratio of high-impact variants among 4,921,131,336 absent, 62,134,299 singleton, 38,474,061 rare (MAF < 0.1%), and 14,438,258 common SNVs in gnomAD as a function of gene LOEUF decile. Genes with a high LOEUF are more tolerant to loss of function. The analysis is restricted to variants within 5 kb of protein-coding genes. The high-impact cutoffs for CADD and AbExp without BCV were set to match the quantile of the high-impact cutoff of AbExp. Error bars show Wald 95% confidence intervals from logistic regression fits.

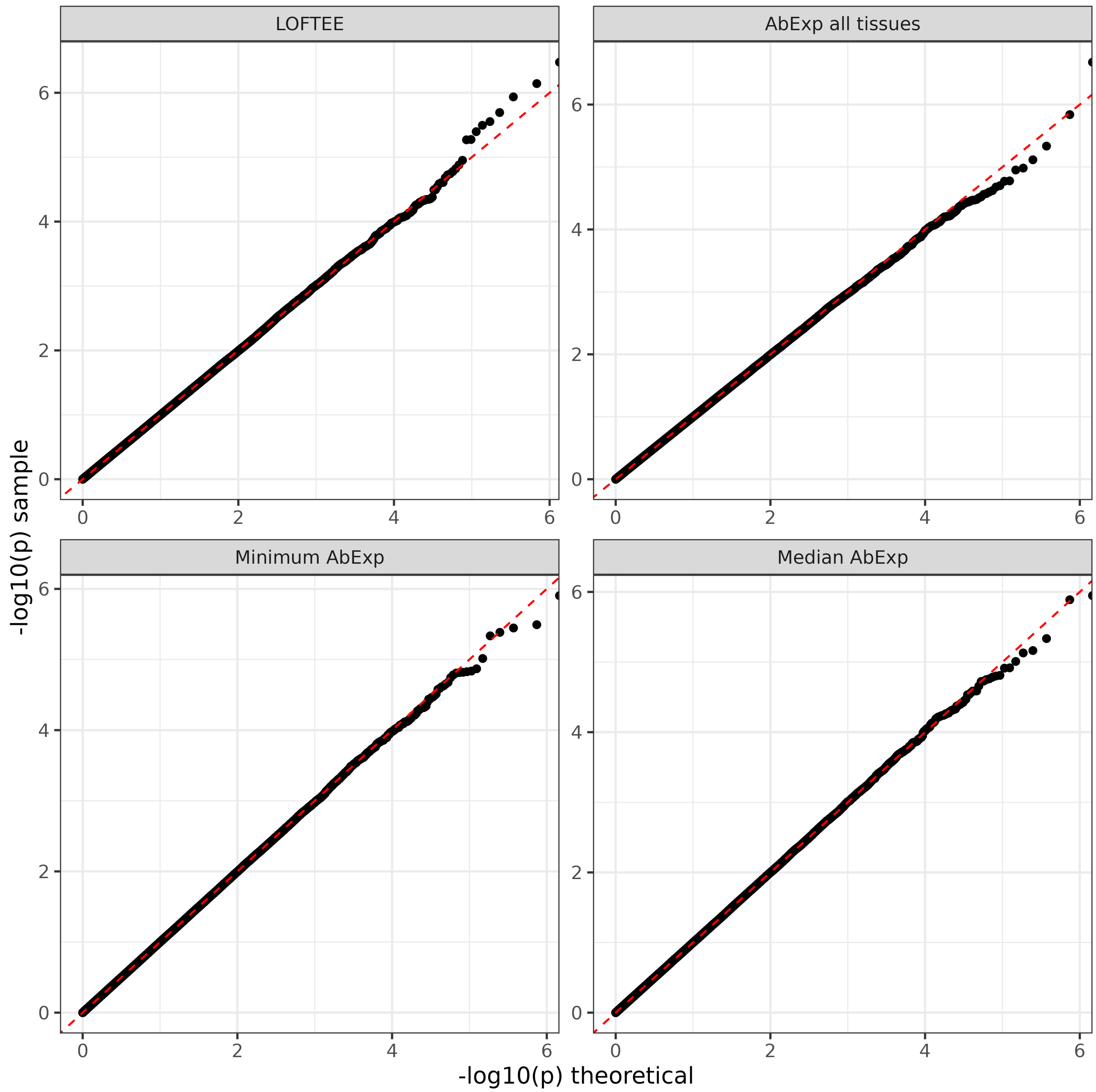

Supplementary Figure 15: Quantile-quantile plot of *p*-values on randomized data against random uniform distribution across all traits facetted by model. *P*-values on randomized data were obtained by shuffling the phenotype randomly once and testing for associations. The data aligns on the diagonal indicating calibration of all four models. Across all four models and 40 traits, we found only two reported associations at FDR < 0.05 on these permuted data, one for AbExp in the “Total bilirubin” trait and one for LOFTEE in the “IGF1” trait.

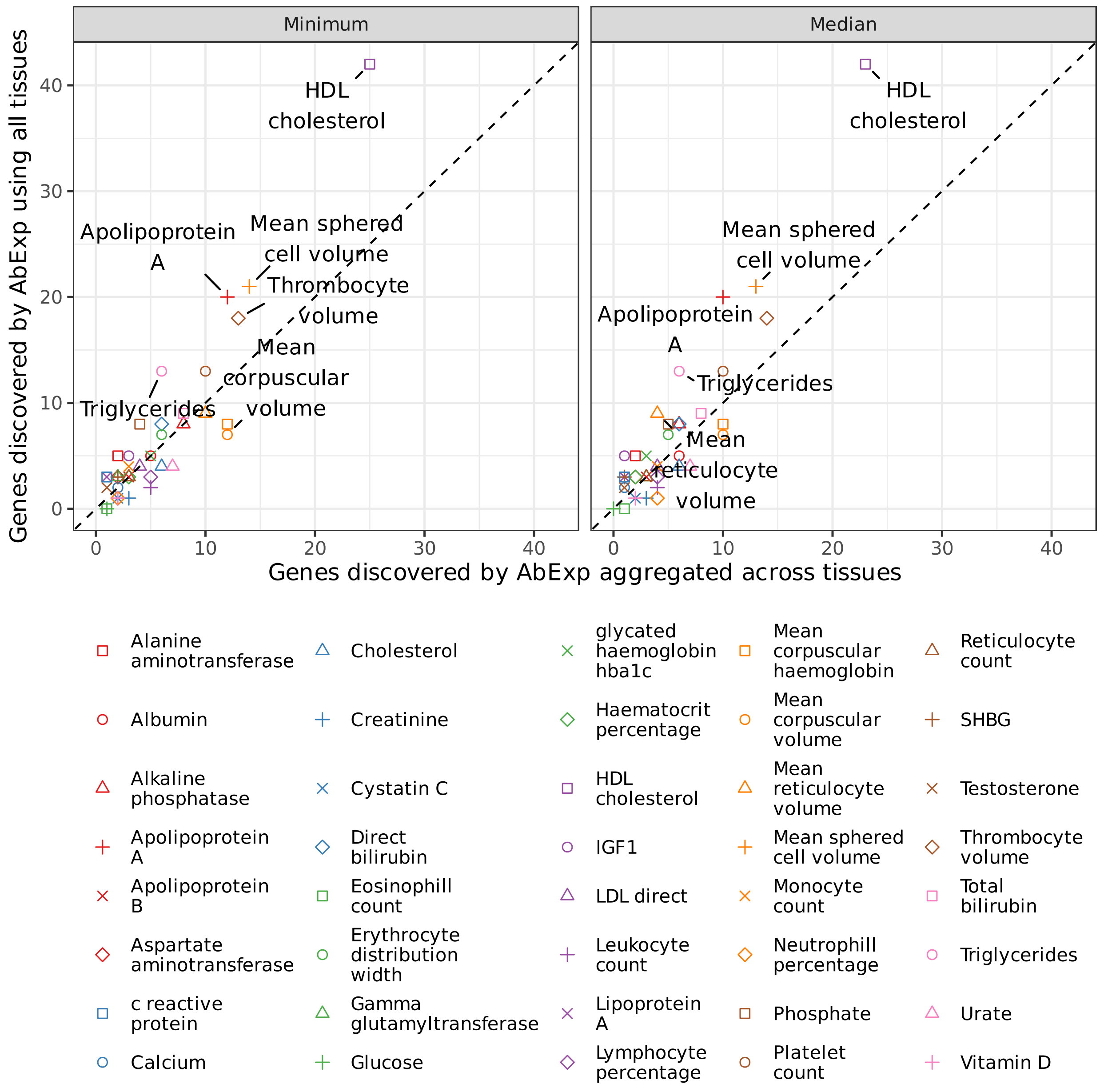

Supplementary Figure 16: **Number of significant blood-trait-associated genes for different AbExp aggregations.** The y-axis shows the number of significantly associated genes when using AbExp scores of all tissues as variables in the regression test. The x-axis shows the corresponding number of significantly associated genes when using an aggregated AbExp score as variable in the regression test. ‘Minimum’ is using the minimum predicted score across tissues per gene, ‘Median’ uses the median score across tissues.

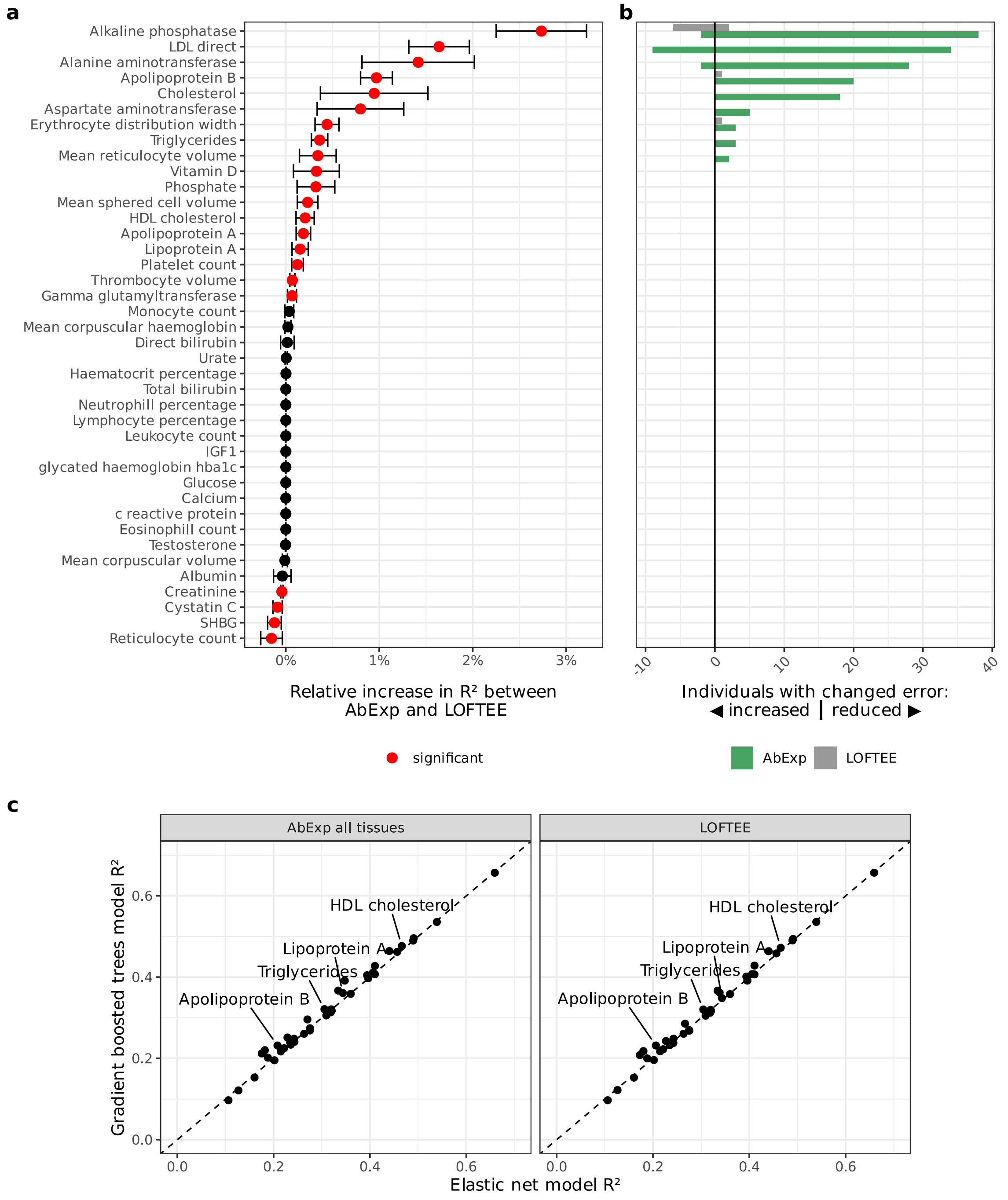

Supplementary Figure 17: **AbExp improvements over LOFTEE in phenotype prediction also hold with elastic-net based predictions. (a)** Relative R^2 increase between AbExp-based and LOFTEE-based predictions across traits. Traits with a significant difference between both models are marked red (two-sided paired t-test, nominal $P < 0.05$). Error bars show the standard deviation among 5 cross-validation folds. **(b)** Positive bars show the number of individuals with an error reduced by at least one standard deviation in the trait scale and therefore improved prediction, negative bars show the number of individuals with an error increased by at least one standard deviation in the trait scale and therefore worse prediction of the AbExp-based model (green) and the LOFTEE-based model (grey). **(c)** R^2 of gradient boosted trees models against R^2 of elastic net models across traits, when using AbExp scores or LOFTEE. All data presented in this figure are computed on held-out folds of a 5-fold cross-validation within a third of the UKBB data.

Supplementary Tables

	Individuals	Genes	Tissues	Samples	Underexpressed outliers	Non-outliers	Overexpressed outliers
**Unfiltered**	946	33615	49	16213	27399	285256273	44987
**Samples with whole genomes**	633	33615	49	11215	19051	197083281	31637
**Keep only protein-coding genes**	633	18563	49	11215	15320	147883919	26338
**Remove samples with many outliers**	633	18563	49	10999	13873	145015257	20047
**Keep only genes of samples that have a sufficiently large expected number of reads ('mu' > 450)**	633	18171	49	10999	11200	99434253	14464

Supplementary Table 1: **GTEx dataset filtering**

	Individuals	Genes	Tissues	Samples	Underexpressed outliers	Non-outliers	Overexpressed outliers
**Unfiltered**	325	14740	1	325	1681	4787910	909
**Samples with whole genomes**	311	14740	1	311	1626	4581633	881
**Keep only protein-coding genes**	311	11849	1	311	1471	3682880	688
**Remove samples with many outliers**	295	11849	1	295	983	3493975	497
**Keep only genes of samples that have a sufficiently large expected number of reads ('mu' > 450)**	295	11314	1	295	808	2586111	384

Supplementary Table 2: **Mito-disease dataset filtering**

	Individual s	Genes	Tissue s	Sample s	Underexpress ed outliers	Non-outliers	Overexpress ed outliers
**Unfiltered**	253	16381	1	253	1442	4142056	895
**Keep only protein-coding genes**	253	12771	1	253	1231	3229015	817
**Remove samples with many outliers**	233	12771	1	233	811	2974445	387
**Keep only genes of samples that have a sufficiently large expected number of reads ('mu' > 450)**	233	11748	1	233	653	2182453	302

Supplementary Table 3: **ALS dataset filtering**
