# Supplementary material for: Aberrant gene expression prediction across human tissues
Source: Nat Commun. 2025 Mar 29;16:3061. doi: 10.1038/s41467-025-58210-w (PMC11954926; doi:10.1038/s41467-025-58210-w)
Supplement: Supplementary file 2 — Description of Additional Supplementary Files [file 41467_2025_58210_MOESM2_ESM.pdf]

Description of Additional Supplementary Files

File Name: Supplementary Data 1

Description: Blood traits PRS score mapping

Mapping of blood traits to polygenic risk scores from the PGS catalog and Genebass phenotype codes

File Name: Supplementary Data 2

Description: RVAT results

List of significant gene-trait associations found with AbExp and LOFTEE
