# Supplementary material for: Aberrant gene expression prediction across human tissues
Source: Nat Commun. 2025 Mar 29;16:3061. doi: 10.1038/s41467-025-58210-w (PMC11954926; doi:10.1038/s41467-025-58210-w)
Supplement: Supplementary file 5 — Transparent Peer Review file [file 41467_2025_58210_MOESM5_ESM.pdf]

nature portfolio

Peer Review File

Aberrant gene expression prediction across human tissues

Corresponding Author: Professor Julien Gagneur

This manuscript has been previously reviewed at another journal. This document only contains information relating to versions considered at Nature Communications.

Version 0:

Reviewer comments:

Reviewer #1

(Remarks to the Author)
The authors have adequately addressed our comments from the initial review. They have provided thorough and thoughtful revisions that sufficiently resolve the points of discussion, including justifying thresholds chosen in their methods and generating precision-recall curves for AbExp and models used in benchmarking.

We have reviewed the updated manuscript and supplemental materials and find no residual concerns that require further clarification. The manuscript represents a meaningful contribution to the field of variant effect prediction, and we recommend it for publication with minimal further edits.

(Remarks on code availability)

Reviewer #2

(Remarks to the Author)
I thank the authors for revising the manuscript. My original points have been addressed.

(Remarks on code availability)

Open Access This Peer Review File is licensed under a Creative Commons Attribution 4.0 International License, which permits use, sharing, adaptation, distribution and reproduction in any medium or format, as long as you give appropriate credit to the original author(s) and the source, provide a link to the Creative Commons license, and indicate if changes were made.
In cases where reviewers are anonymous, credit should be given to 'Anonymous Referee' and the source.
The images or other third party material in this Peer Review File are included in the article's Creative Commons license, unless indicated otherwise in a credit line to the material. If material is not included in the article's Creative Commons license and your intended use is not permitted by statutory regulation or exceeds the permitted use, you will need to obtain permission directly from the copyright holder.
To view a copy of this license, visit https://creativecommons.org/licenses/by/4.0/
